# Analysis and therapeutic schedule of the postoperative recurrence of bone tuberculosis

**DOI:** 10.1186/1749-799X-8-47

**Published:** 2013-12-17

**Authors:** Lei Yang, Zhonghe Liu

**Affiliations:** 1 School of Nursing, Xinxiang Medical University, Xinxiang 453003, China; 2 Department of Orthopedics, The First Affiliated Hospital of Xinxiang Medical University, Weihui, Henan 453100, China

**Keywords:** Bone tuberculosis, Recurrence, Reoperation, Therapeutic schedule

## Abstract

**Objective:**

The present study aims to analyze the main reasons that lead to the failure of bone tuberculosis (TB) surgery and the efficacy of reoperation.

**Methods:**

A total of 3,000 cases of bone TB patients were examined retrospectively. Of these, 180 cases had recurrence, including 135 cases of spinal TB and 45 cases of limb TB. Preoperative indicators of duration of anti-TB chemotherapy, nutritional conditions, temperature conditions and erythrocyte sedimentation rate, and medication time of postoperative and recurrence were statistically analyzed.

**Results:**

Of all 180 cases with reoperation, 176 cases were cured, and four paralyzed patients were symptomatically improved. The causes of postoperative recurrence of bone TB were relatively complex. Efficacy of reoperation was evaluated. Shorter chemotherapy duration, long-term illness, poor health, a higher body temperature, and an accelerated ESR are likely to increase the risk of recurrence.

**Conclusions:**

Given the operation failure, careful analysis of the failure reasons and the targeted reoperation can obtain satisfactory results, thereby avoiding the failure of the initial surgery.

## Introduction

Tuberculosis (TB), which is one of the major infectious diseases in the world today, has caused a huge threat to human health. About one third of the human population is infected with mycobacterium TB, with about 800 million new infections and up to two million deaths caused by TB each year. With the rapid growth of the global population, the acceleration of the population flow, and the human immunodeficiency virus (HIV) infection, morbidity of TB has been increasing every year [1-3]. In addition, the threat of drug-resistant TB is increasing. Various clinical resistances to first- and second-line anti-TB drugs have been observed among TB patients, and those with multi-drug resistant TB (MDR-TB) and extensively drug-resistant TB (XDR-TB) are also progressive [4-6]. Lung TB accounts for the vast majority of the proportion of drug-sensitive and drug-resistant TB. Consequently, TB research has focused on lung TB, while those on bone TB are fewer. Bone TB is a chronic disease, which is difficult to treat and easy to recur, and its morbidity has increased in the past 10 years [7,8].

Drugs and surgery are the main treatments for bone TB. A certain recurrence rate occurs after surgery. In this study, 180 bone TB patients who underwent reoperation were studied to provide a basis for clinical treatment of bone TB.

## Materials and methods

### Clinical data

Of all 3,000 cases of bone TB in the First Affiliated Hospital of Xinxiang Medical College from January 2005 to December 2011, 180 cases had recurrence, of which 108 were male and 72 female. Their ages ranged from 23 to 63 years, and average age was 40 ± 10.2 years. Spinal TB was found in 135 cases and limb TB in 45 cases. Local excision, decompression, bone grafting, and internal fixation, as well as compression arthrodesis were performed as the initial surgery. This study was conducted in accordance with the declaration of Helsinki. This study was conducted with approval from the Ethics Committee of the First Affiliated Hospital of Xinxiang Medical University (permit number: 20060828001). Written informed consent was obtained from all participants.

### Data analysis

Indicators of 180 patients with bone TB were analyzed retrospectively. These indicators included duration of anti-TB chemotherapy, nutritional conditions, body temperature, and erythrocyte sedimentation rate (ESR) before operation, as well as medication time and recurrence after operation.

### Operation methods

Routine physical examination and blood, urine, and liver function tests were carried out before the surgery to learn the medical history of and causes for reoperation of the patients. Through conventional anti-TB treatment, surgery can be performed when ESR and C-reactive protein decreased. Effective preoperative communication between doctors and patients was necessary for the establishment of trust with each other. Additionally, sufficient amount of blood was essential for surgical bleeding.

Therapeutic schedules were made in accordance with the causes for reoperation and their specific conditions. Simple local excision or combined decompression, bone grafting, and internal fixation were chosen for spinal TB, whereas simple local excision or combined compression arthrodesis for limb TB. The sequestra, necrotic tissue, invaded intervertebral disc, and pus were cleared to the greatest extent. Supplementary blood was administered according to blood loss in patients during surgery.

The recovery status and supply of nutrients of patients, especially neurotrophic supply, and traits of the drainage were taken into intensive care and postoperative surveillance for timely targeted treatment.

## Results

### Analysis of the causes of reoperations

Causes of reoperation were complex through the analysis of patients with bone TB. Generally, selective operation was carried out after 4 weeks of anti-TB chemotherapy. Of all the patients, 72.2% received operations after 1 week of administration, 17.2% in 2 weeks, and 10.6% in 4 weeks, demonstrating that the risk of recurrence increased with shortened chemotherapy duration. Of all the patients, 85.0% were administered anti-TB chemotherapy for less than 1 year after operation, demonstrating that the risk of recurrence increased with shorter postoperative chemotherapy duration. Higher body temperature was found in 72.8% of the cases, which demonstrated that the risk of recurrence increased with a higher temperature. An accelerated ESR was found in 76.7% of the patients, thereby demonstrating that the risk of recurrence was related with an accelerated ESR (Tables 1, 2, 3, 4).

**Table 1:** Influence of preoperative anti-TB chemotherapy on recurrence of bone tuberculosis

**Duration of preoperative anti-TB chemotherapy (week)**	**Spinal tuberculosis ( *n *)**	**Limb tuberculosis ( *n *)**	**Total**	**Ratio (%)**
1	96	34	130	72.2
1 ~ 2	23	8	31	17.2
2 ~ 4	17	2	19	10.6

**Table 2:** Influence of postoperative medication on recurrence of bone tuberculosis

**Duration of postoperative medication (month)**	**Spinal tuberculosis ( *n *)**	**Limb tuberculosis ( *n *)**	**Total**	**Ratio (%)**
6	45	22	67	37.2
8	67	19	86	47.8
12	23	4	27	15.0

**Table 3:** Influence of body temperature on recurrence of bone tuberculosis

**Body temperature**	**Spinal tuberculosis ( *n *)**	**Limb tuberculosis ( *n *)**	**Total**	**Ratio (%)**
Normal	37	12	49	27.2
37°C ~ 37.4°C	48	15	63	35.0
37°C ~ 38°C	42	17	59	32.8
Above 38°C	28	1	29	16.1

**Table 4:** Influence of ESR on recurrence of bone tuberculosis

**ESR**	**Spinal tuberculosis ( *n *)**	**Limb tuberculosis ( *n *)**	**Total**	**Ratio (%)**
Accelerated	113	25	138	76.7
Normal	22	20	42	23.3

Long-term illness, food preference, and poor health were likely to increase the risk of recurrence. Bone destruction accounted for 8.8% of all kinds of recurrent manifestations, abscess in 74.4%, and fistula in 16.7%, demonstrating that cold abscess was more common (Tables 5 and 6).

**Table 5:** Nutritional status

**Nutritional status**	**Spinal tuberculosis ( *n *)**	**Limb tuberculosis ( *n *)**	**Total**	**Ratio (%)**
Abstention from meat	118	32	150	83.3
Abstention from vegetables	17	13	30	16.7

**Table 6:** Symptoms of recurrence

**Symptoms of recurrence**	**Spinal tuberculosis ( *n *)**	**Limb tuberculosis ( *n *)**	**Total**	**Ratio (%)**
Bone destruction	9	7	16	8.8
Abscess	112	22	134	74.4
Fistula	14	16	30	16.7

### Result of reoperation

A total of 180 patients underwent reoperation. The preoperative factors included careful analysis of the causes and the cautious selection of the operation time. Corrosion near the vertebral edge, annulus fibrosus in the intervertebral disc, and residual TB products in the contralateral lesion beyond direct vision were carefully removed. With reoperation, 176 cases were cured, and four paralyzed patients were symptomatically improved.

## Discussion

The recurrence of bone TB was related to many factors through the treatment of 180 recurrent patients. The anti-TB chemotherapy of quadruple or quintuple anti-TB drugs was administrated as early as possible, and efficacy was observed. Invalid anti-TB drugs should promptly be adjusted. In case of liver function damage, the dosage was timely reduced in patients with poor health, old age, and children after 5 to 7 days of treatment with high-dose anti-TB drugs [9,10]. Surgical treatment without anti-TB chemotherapy was prohibited for extremely high risk of recurrence. Operation was recommended after 4 weeks of anti-TB treatment, which was prolonged on the condition that temperature was above 38°C and ESR was above 50 mm/h [11].

The eating habits of the persons were adjusted to a high-calorie, high-protein, high-vitamin, high-carbohydrate, and appropriate fat food. Most patients have lost their appetite for meat, and a few cases had no appetite for vegetables. More beef, lamb, pork, fish, fresh vegetables, and fruits were beneficial for the chronic wasting disease TB [12,13].

Emotional adjustment in patients was also important for patients with depression and irritability due to long-term illness and poverty. Patient explanation for the cause, diagnosis, treatment, and prevention of TB was significant for confidence enhancement and better cooperation among patients.

Careful analysis of the condition of patients and favorable surgical schedule were taken prior to surgery. Sequestra, invaded intervertebral disc, necrotic tissue, corrosion near the vertebral edge, unilateral local excision, contralateral abscess, gravitation abscess (if it was present), and annulus fibrosus in the intervertebral disc were all thoroughly removed without any residue. Thoroughness of the surgery is the key factor that influences the success of TB treatment [14,15].

In conclusion, TB is characterized by difficulty of treatment and easy recurrence, yet bone TB can be cured by initial surgery with improved success rate and targeted reoperation. Success rates were improved by the good emotional status of patients, systematic anti-TB chemotherapy and an appropriate time of surgery before operation, a thorough removal of the lesions without any sequestrum, and gravitation abscess in operation, coupled with regular as well as combined and adequate anti-TB chemotherapy after operation.

## Conclusion

The causes of postoperative recurrence of bone TB were relatively complex. A careful analysis of the reasons and the targeted reoperations can obtain satisfactory results, thereby avoiding the failure of the initial surgery.

## Competing interests

The authors declare that they have no competing interests.

## Authors’ contributions

ZL designed the study, and LY performed the study. ZL and LY analyzed the data and wrote the paper. Both authors read and approved the final manuscript.

## References

[B1] Orcau À Caylà JA Martínez JA Present epidemiology of tuberculosis: prevention and control programs Enferm Infecc Microbiol Clin Suppl 2011 8 2 7 10.1016/S0213-005X(11)70011-8 21420560

[B2] Jassal MS Bishai WR Epidemiology and challenges to the elimination of global tuberculosis Clin Infect Dis 2010 8 Suppl 3 S156 S164 20397943 10.1086/651486 PMC4575217

[B3] Nahid P Menzies D Update in tuberculosis and nontuberculous mycobacterial disease 2011 Am J Respir Crit Care Med 2012 8 1266 1270 10.1164/rccm.201203-0494UP 22707733

[B4] Taylor AB Kurbatova EV Cegielski JP Prevalence of anti-tuberculosis drug resistance in foreign-born tuberculosis cases in the U.S. and in their countries of origin PLoS One 2012 8 e49355 10.1371/journal.pone.0049355 23145161 PMC3492290

[B5] Maurya AK Kant S Nag VL Kushwaha R Dhole TN Trends of anti-tuberculosis drug resistance pattern in new cases and previously treated cases of extrapulmonary tuberculosis cases in referral hospitals in northern India J Postgrad Med 2012 8 185 189 10.4103/0022-3859.101379 23023350

[B6] Drobniewski F Nikolayevskyy V Balabanova Y Bang D Papaventsis D Diagnosis of tuberculosis and drug resistance: what can new tools bring us? Int J Tuberc Lung Dis 2012 8 860 870 10.5588/ijtld.12.0180 22687497

[B7] Garg RK Somvanshi DS Spinal tuberculosis: a review J Spinal Cord Med 2011 8 440 454 10.1179/2045772311Y.0000000023 22118251 PMC3184481

[B8] Lesić AR Pesut DP Marković-Denić L Maksimović J Cobeljić G Milosević I Atkinson HD Bumbasirević M The challenge of osteo-articular tuberculosis in the twenty-first century: a 15-year population-based study Int J Tuberc Lung Dis 2010 8 1181 1186 20819266

[B9] Liu Y Chen Y Yang L Zhou X Wang C Qi M Yuan W The surgical treatment and related management for post-tubercular kyphotic deformity of the cervical spine or the cervico-thoracic spine Int Orthop 2012 8 367 372 10.1007/s00264-011-1438-9 22215364 PMC3282862

[B10] Dai LY Jiang LS Wang YR Jiang SD Chemotherapy in anterior instrumentation for spinal tuberculosis: highlighting a 9-month three-drug regimen World Neurosurg 2010 8 560 564 10.1016/j.wneu.2010.02.023 20920943

[B11] Feng L Qu DB Jin DD Chen JT Observation of body temperature and erythrocyte sedimentation rate in spinal tuberculosis patients with anterior interbody autograft and internal fixation Di Yi Jun Yi Da Xue Xue Bao 2002 8 84 85 12390858

[B12] Xiao L Jiang Y Tian Y Li X Yin Q Liu Z Han L Fu J Primary anterior focus debridement and bone autograft with internal fixation via transperitoneal approach for tuberculosis of lumbosacral junction Zhongguo Xiu Fu Chong Jian Wai Ke Za Zhi 2009 8 913 916 19728604

[B13] Shen HL Xia Y Li P Wang J Han H Arthroscopic operations in knee joint with early-stage tuberculosis Arch Orthop Trauma Surg 2010 8 357 361 10.1007/s00402-009-0881-1 19387669

[B14] Zhang HQ Li JS Zhao SS Shao YX Liu SH Gao Q Lin MZ Liu JY Wu JH Chen J Surgical management for thoracic spinal tuberculosis in the elderly: posterior only versus combined posterior and anterior approaches Arch Orthop Trauma Surg 2012 8 1717 1723 10.1007/s00402-012-1618-0 23053192

[B15] Wang XT Zhou CL Xi CY Sun CL Yan JL Surgical treatment of cervicothoracic junction spinal tuberculosis via combined anterior and posterior approaches in children Chin Med J (Engl) 2012 8 1443 1447 22613651

